# 645. Trends in the Susceptibility of US *Acinetobacter baumannii-calcoaceticus* Species Complex and *Stenotrophomonas maltophilia* Isolates to Minocycline, 2014-2021

**DOI:** 10.1093/ofid/ofac492.697

**Published:** 2022-12-15

**Authors:** Dee Shortridge, Cecilia G Carvalhaes, Jennifer M Streit, Mariana Castanheira

**Affiliations:** JMI Laboratories, North Liberty, Iowa; JMI Laboratories, North Liberty, Iowa; JMI Laboratories, North Liberty, Iowa; JMI Laboratories, North Liberty, Iowa

## Abstract

**Background:**

*Acinetobacter baumannii-calcoaceticus* species complex (ACB) and *Stenotrophomonas maltophilia* (SM) are opportunistic, non-fermentative organisms that can cause serious hospital-acquired infections in immunocompromised patients. These pathogens are inherently resistant to several common drug classes and often acquire other resistance mechanisms, making them difficult to treat. In this study, we analyzed the susceptibility of contemporary ACB and SM isolates to minocycline (MIN), levofloxacin (LEV), meropenem (MER) for ACB, and trimethoprim-sulfamethoxazole (T/S) for SM. Isolates were collected as a part of the SENTRY Antimicrobial Surveillance Program from 2014-2021.

Susceptibilities of SM and ACB to MIN and comparators, 2014-2021

**Figure:**
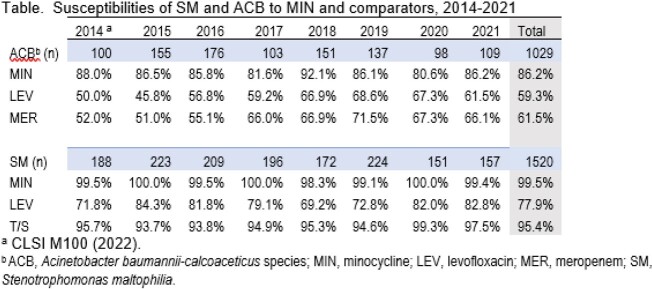

**Methods:**

Isolates were collected from hospitalized patients in 35 US medical centers. Hospitals submitted 1 isolate per patient per infection episode that met local criteria for being the likely causative pathogen. Identification was performed by the submitting laboratory and confirmed by JMI Laboratories with matrix-assisted laser desorption ionization-time of flight mass spectrometry or other standard methods as required. Isolates were tested for susceptibility (S) to MIN and comparators using the CLSI broth microdilution method. All infection types were included in the analysis. CLSI (2022) breakpoints were applied.

**Results:**

A total of 1,029 ACB and 1,520 SM were tested. Pneumonia in hospitalized patients was the most common infection from which ACB (57.0%) and SM (73.9%) were isolated. The %S of the agents tested against the organisms in this study are shown in the table. MIN had the highest %S for ACB (86.2%S) and SM (99.5%S). The %S of ACB and SM to all 3 agents varied over the period studied. MIN %S to ACB decreased in 2020 (80.6%) but rebounded in 2021 (86.2%). LEV and MER showed an overall trend of increasing S for ACB, with slightly lower %S in 2020-2021. SM had stable %S to MIN and T/S ( >98.3% and >93.7%, respectively). LEV varied from 84.3%S (2015) to 69.2%S (2018).

**Conclusion:**

%S to MIN remained stable and higher than other agents tested for both ACB and SM, pathogens which have limited therapeutic alternatives. ACB showed < 6% decrease in %S to all 3 agents in 2020-2021. These *in vitro* data suggest that MIN is a useful treatment option for infections caused by ACB or SM.

**Disclosures:**

**Dee Shortridge, PhD**, AbbVie: Grant/Research Support|JMI Laboratory: Employee|Melinta: Grant/Research Support|Menarini: Grant/Research Support|Shionogi: Grant/Research Support **Cecilia G. Carvalhaes, MD, PhD**, AbbVie: Grant/Research Support|Cidara: Grant/Research Support|Melinta: Grant/Research Support|Pfizer: Grant/Research Support **Jennifer M. Streit, BS, MT(ASCP)**, Cidara: Grant/Research Support|GSK: Grant/Research Support|Melinta: Grant/Research Support|Shionogi: Grant/Research Support **Mariana Castanheira, PhD**, AbbVie: Grant/Research Support|Cidara: Grant/Research Support|GSK: Grant/Research Support|Melinta: Grant/Research Support|Pfizer: Grant/Research Support|Shionogi: Grant/Research Support.

